# METH-2 silencing and promoter hypermethylation in NSCLC

**DOI:** 10.1038/sj.bjc.6602107

**Published:** 2004-08-24

**Authors:** J R Dunn, D Panutsopulos, M W Shaw, J Heighway, R Dormer, E N Salmo, S G Watson, J K Field, T Liloglou

**Affiliations:** 1 Roy Castle Lung Cancer Research Programme, University of Liverpool Cancer Research Centre, 200 London Road, Liverpool L3 9TA, UK; 2 University of Crete Medical School, Virology Laboratory, Heraklion, Greece; 3 St Helens & Knowsley Hospitals NHS Trust, Warrington Road, Prescot, Liverpool L35 5DR, UK

**Keywords:** angiogenesis, lung cancer, METH-2, methylation

## Abstract

The antiangiogenic factor METH-2 (ADAMTS-8) was identified in a previous dual-channel cDNA microarray analysis to be at least two-fold under-represented in 85% (28 out of 33) of primary non-small-cell lung carcinomas (NSCLCs). This observation has been validated in an independent series of NSCLCs and adjacent normal tissues by comparative multiplex RT—PCR, and METH-2 mRNA expression was dramatically reduced in all 23 tumour samples analysed. Immunohistochemical analysis of the same sample set demonstrated that METH-2 was strongly expressed in 14 out of 19 normal epithelial sites examined but only one out of 20 NSCLCs. DNA methylation analysis of the proximal promoter region of this gene revealed abnormal hypermethylation in 67% of the adenocarcinomas and 50% of squamous cell carcinomas, indicating that epigenetic mechanisms are involved in silencing this gene in NSCLC. No homozygous deletions of METH-2 were found in lung cancer cell lines. Allelic imbalance in METH-2 was assessed by an intronic single nucleotide polymorphism (SNP) assay and observed in 44% of informative primary samples. In conclusion, the downregulation of METH-2 expression in primary NSCLC, often associated with promoter hypermethylation, is a frequent event, which may be related to the development of the disease.

The ability of a tumour to grow more than 2–3 mm^3^ relies on the creation of a new blood supply, which facilitates growth by allowing the delivery of nutrients and oxygen to the new tissue (Folkman and Cotran, 1976; Folkman *et al*, 1989). This process, angiogenesis, is the formation of new blood vessels from the existing vasculature, more specifically from the vascular endothelial cells. Tumour angiogenesis involves a number of factors that may be paracrine from the adjacent tumour, stromal cells, inflammatory cells, extracellular matrix (ECM), and/or autocrine from the endothelial cells themselves. Antiangiogenesis approaches are considered to be possible adjuncts to conventional therapy in the treatment of lung cancer patients.

METH-2 (ADAMTS-8) is a member of the ADAMTS family (a disintegrin and metalloprotease with thrombospondin-1-like motifs). This family of genes shows significant sequence similarity to snake venom metalloprotease and disintegrin. METH-2 was fully characterised by Vazquez *et al* (1999) along with its counterpart METH-1 (ADAMTS1). METH-2 is expressed in various human tissues, exhibiting high levels in the adult and fetal normal lung. It is a single-copy gene, and encodes a proteolytically processed protein. METH-2 protein has a more powerful antiangiogenic effect than thrombospondin-1 or endostatin, and can specifically suppress endothelial cell proliferation (Vazquez *et al*, 1999). Human METH-2 is located at 11q25 (Georgiadis *et al*, 1999).

The role of METH genes in cancer development is largely unclear. Masui *et al* (2001) have reported that METH-1 appears to be involved in the progression of pancreatic cancer while METH-2 was not expressed. To date, to our knowledge, there is no specific report on the involvement of these genes in lung cancer. However, METH-2 was highlighted in our previous study (Heighway *et al*, 2002) in which a panel of cDNA microarrays comprising 47 650 transcript elements was used to analyse gene expression patterns in 39 ressected primary human non-small-cell lung tumours that were compared to paired normal lung tissues. These data demonstrated that METH-2 was significantly under-represented (two-fold or lower) in 85% (28 out of 33) of the analysed non-small-cell tumours. This suggested the possibility that the loss of METH-2 function might be an important factor in tumour angiogenesis. In this study, we have validated our previous microarray observations of METH-2 expression in an independent series of non-small-cell lung carcinomas (NSCLCs) and we have investigated possible genetic and epigenetic mechanisms responsible for this inactivation.

## MATERIALS AND METHODS

### Tissues and cell lines

In all, 12 adenocarcinomas (ADCs) and 11 squamous cell carcinomas (SCCs) were selected from our tumour bank, along with their paired normal tissues. All these tumours had a T stage=2 while the N stage varied (Table 1

**Table 1:** METH-2 expression and methylation results in relation to clinicopathological parameters

						**mRNA expression**		**Protein expression**			**Methylation**	
**Hist. no.**	**Age**	**Histology**	**Differentiation**	**T status**	**N status**	**N**	**T**	**N_adj_**	**N_T_**	**T**	**N**	**T**
L124	65	AdC	Poor	2	0	+	↓	NA	NA	NA	−	+
L298	68	AdC	Poor	2	2	+	↓	Strong/C	NA	Reduced/B	−	−
L308	68	AdC	Poor	2	0	+	↓	NA	NA	Reduced/B	−	+
L314	52	AdC	Poor	2	2	+	↓	Strong/B	NA	Reduced/B	−	+
L323	74	AdC	Poor	2	2	+	↓	Reduced/A	Strong/B	Negative	−	+
L326	53	AdC	Moderate	2	0	+	↓	Strong/B	Reduced/C	Negative	−	−
L336	73	AdC	Poor	2	1	+	↓	Reduced/B	NA	Negative	−	−
L341	58	AdC	Moderate	2	0	+	↓	Negative	Strong/B	Reduced/B	−	−
L352	61	AdC	Poor	2	1	+	↓	NA	Reduced/C	Reduced/C	+	+
L353	67	AdC	Poor	2	1	+	↓	Strong/C	NA	Negative	−	+
L358	68	AdC	Poor	2	2	+	↓	NA	Strong/C	Reduced/B	−	−
L378	55	AdC	Poor	2	1	+	↓	Strong/B	Strong/C	Negative	−	+
L112	74	SqCC	Moderate	2	0	+	↓	NA	NA	Negative	−	−
L144	61	SqCC	Moderate	2	0	+	↓	NA	NA	Negative	−	−
L151	70	SqCC	Poor	2	1	+	↓	NA	NA	Negative	−	−
L152	56	SqCC	Well	2	1	+	↓	NA	Strong/C	Reduced/C	−	−
L153	67	SqCC	Moderate	2	0	+	↓	NA	NA	Reduced/A	−	+
L161	76	SqCC	Moderate	2	0	+	↓	NA	Strong/C	Negative	−	+
L177	71	SqCC	Moderate	2	0	+	↓	NA	NA	Negative	−	+
L180	45	SqCC	Well	2	1	+	↓	Strong/B	NA	NA	−	−
L362	52	SqCC	Moderate	2	1	+	↓	NA	NA	NA	−	−
L371	60	SqCC	Moderate	2	1	+	↓	Strong/C	NA	Reduced/A	−	−
L373	69	SqCC	Moderate	2	0	+	↓	Strong/B	NA	Strong/B	−	−

Histology: AdC=adenocarcinoma, SqCC=squamous cell carcinoma. mRNA expression: +=METH-2 transcript equally detectable with control gene, ↓=reduced METH-2 transcript. Protein expression: N_adj_=adjacent normal, N_T_=normal within tumour section, T=tumour. Protein expression, staining score: A=less than 5% of cells, B=less than 50%, C=greater than 50%, NA=no available data.

). The mean age of the patients was 64. All 23 tissue pairs were used for examining the expression of METH-2. DNA from 27 additional NSCLC samples (15 SCC, 12 ADC) was used in the methylation analysis. Microscopic examination of H&E-stained sections was carried out to confirm that tumour tissue sections contained >70% tumour cells.

The following nine lung cancer cell lines obtained from American Type Culture Collection (Rockville, MD, USA) were used in the homozygous deletion screening: *squamous*: CRL5802, HTB182, SKMES, SKMES1, LUDLU1; *adenocarcinoma*: A549, SKLU1; *small cell*: DMS53; *large cell*: CORL23.

### DNA extraction, RNA extraction and cDNA synthesis

Genomic DNA from cell lines and the set of 27 unpaired tumours was extracted as previously described (Liloglou *et al*, 2000; Heighway *et al*, 2003). Total RNA and genomic DNA from the 23 normal–tumour paired samples was extracted from 20 × 40 *μ*m frozen tissue sections using the Qiagen® RNA/DNA extraction kit, following the manufacturer's protocol. RNA was aliquotted into 1–2 *μ*g amounts for subsequent cDNA synthesis in 20 *μ*l reactions using the Promega Reverse Transcription System, using oligo-dT primers. RNA aliquots were stored at –80^o^C; cDNAs and genomic DNAs were stored at −20°C.

### Comparative multiplex RT–PCR

To compare the relative expression levels of METH-2 in multiple paired normal and tumour lung cancer tissues, multiplex RT–PCR reactions were set up as described. Two primer sets were used in the same PCR reaction: METH-2 primers spanning exons 3 and 4 (forward: 5′-AAC AAA AGC TGC TCC GTG AT-3′; reverse: 5′-TCT GGT TCA GGT GGA CGA AC-3′) and a control gene primer set (control gene – O28, KIAA0228: amyloid beta precursor protein-binding protein, acc. no. D86981) that had previously been found to be expressed at a constant level in normal and lung cancer tissues (Heighway *et al*, 2002; forward: 5′-GAA CTG TGT GCA CTC CTA TTT G-3′; reverse: 5′-CCG TGC CAA ATA CAC TGC ATG GT-3′). PCR reactions (50 *μ*l) were set up, which contained 1 *μ*l of cDNA, 5 *μ*l of 10 × buffer, 0.5 U of Roche *Taq* polymerase, 1 *μ*l dNTPs (5 mM), 1 *μ*l of O28 primer set (10 pM *μ*l^−1^), 10 *μ*l of METH-2 primer set (10 pM *μ*l^−1^) and 32.75 *μ*l ddH_2_O. cDNA was amplified under the following conditions: 95°C for 5 min, followed by 30 cycles of 94°C for 1 min, 59°C for 30 s, 72°C for 30 s and a final extension step of 72°C for 10 min. Finally, 5 *μ*l of the resulting PCR product was visualised by electrophoresis through a 3% agarose gel containing ethidium bromide.

### Homozygous deletion screening in lung cancer cell lines

To determine whether the chromosomal region containing the METH-2 gene was deleted in lung cancer, 100 ng of genomic cell line DNA was amplified by PCR using METH-2 primers located within exon 2 (forward: 5′-TGC GTA ACT TCT GCA ACT GG-3′) and intron 2 (reverse: 5′-CTT TGA TCT GCC CAT CCT GT-3′). PCR mix was composed of 1 *μ*l DNA, 5 *μ*l 10 × buffer, 0.5 U of Roche *Taq* polymerase, 1 *μ*l dNTPs (5 mM), 1 *μ*l primers (10 pmol *μ*l^−1^) and 32.75 *μ*l ddH_2_O. The reaction profile was 95°C for 5 min, followed by 30 cycles of 94°C for 1 min, 58°C for 30 s, 72°C for 30 s and a final extension of 72^o^C for 10 min. Finally, 5 μl of PCR product was visualised by electrophoresis through a 1% agarose gel containing ethidium bromide.

### Methylation analysis

#### Sodium bisulphite treatment of DNA

A 2 *μ*g portion of genomic DNA was digested with 20 U of *Hind*III in a total volume of 50 *μ*l for 6 h at 37°C and subsequently denatured by adding NaOH to a concentration of 0.3 M and incubating at 42°C for 20 min. A saturated sodium bisulphite solution was made by the addition of 5.4 g sodium metabisulphite (Sigma S-9000) and 0.01 g hydroquinone (Sigma H-9003) in 10 ml of distilled water. pH was brought to 5.0 with NaOH. A 950 *μ*l volume of the resulting solution was added to the DNA samples, which were then incubated at 55°C for 16 h.

DNA was recovered using Wizard® DNA Clean-Up System (Promega, UK) following the manufacturer's protocol. Desulphonation was achieved by the addition of NaOH to a concentration of 0.3 M and incubation at 37°C for 30 min. DNA was precipitated with ammonium acetate/ethanol, recovered by centrifugation and eluted in 50 ml of 1 mM Tris-HCl and 0.1 mM EDTA (pH 8.0). DNA samples were stored at −20°C until use.

#### Competitive methylation-specific PCR

We have designed a competitive methylation-specific PCR (cMSP) assay by combining methylation-specific primers (forward: 5′-CGCGGTATAGGTTGATCGTC-3′; reverse: 5′-GTACTACGCCTAACGCCCG-3′) that lie in the CpG island located in exon 1 of METH-2 and methylation-independent primers (forward: 5-TTGATTGGGGTTTGAGAGGATT-3; reverse: 5′-CCCAACTAACCACACTCCAAACT-3′) that anneal in intron 3 of the gene. The PCR mix was composed of 5 *μ*l 2 × QIAGEN Multiplex PCR Master Mix (Qiagen, UK), 0.35 pmol control primers, 5 pmol methylation-specific primers and 2 *μ*l of bisulphite-treated DNA. The reaction profile was 95°C for 15 min followed by 36 cycles consisting of 94°C for 30 s, 60°C for 40 s, 72°C for 70 s and a final extension of 72°C for 20 min. PCR products (control: 299 bp; methylation-specific: 169 bp) were analysed on agarose gels as well as chip capillary electrophoresis (Agilent 2100 Bioanalyser).

### Allelic imbalance analysis at the METH-2 locus

Allelic imbalance (AI) in METH-2 was assessed in 23 normal/tumour pairs using an intragenic, intronic single nucleotide polymorphism (SNP): rs1552330 (NCBI). SNP templates were generated by PCR using the same reaction mix and reaction profile used for homozygous deletion screening. PCR primers were as follows: forward, 5′-ATGGAGTCTTCCCAGGTGGT-3′; reverse, 5′-TGCCAAAGCTGGTCTCACTA-3′. A 10 *μ*l volume of PCR template was then digested with 5 U *Bst*UI in a total volume of 30 *μ*l for 3 h at 60°C. A 20 *μ*l portion of the digested template was visualised by electrophoresis through a 3% agarose gel containing ethidium bromide. Allelic imbalance was visually assessed as an imbalance of the band (digested-to-undigested) ratio in comparison to the normal counterpart.

### Immunohistochemical analysis of METH-2

Immunohistochemical (IHC) analysis was performed on 32 × 5 *μ*m formalin-fixed, paraffin-embedded sections with METH-2 antibody (1 : 500 dilution) (METH-2 AB-1, Oncogene Research Products; cat#PC508). Primary antibody was diluted in DAKO ChemMate™ antibody diluent containing blocking protein (code no. S 2022). Each run comprised tumour lung tissue sections with paired normal when available, a positive control tissue section (normal human stomach or sigmoid colon – previously determined using a tissue microarray containing 35 different human tissue types) and a negative control (no primary antibody) for each of these sections. Tissues were first dewaxed, followed by endogenous peroxidase blocking in 3% peroxide solution. Tissues were then subjected to antigen retrieval by microwaving for 15 min in antigen unmasking solution (Vector Laboratories Inc., Burlingame, CA 94010, USA; cat#H-3300), then stained for 1 h with METH-2 primary antibody. Tissues were incubated with a biotinylated anti-rabbit secondary antibody, followed by streptavidin incubation, then detection with DAB chromogen (DAKO LSAB2 Peroxidase system). Protein expression was scored by two independent pathologists (MS and ES); each section was assessed as to the location, intensity and proportion of cells positive with the antibody. Intensity was defined as negative, weak and strong.

## RESULTS

### METH-2 expression in primary NSCLC

**Figure 1:**
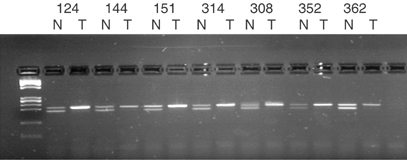
Relative expression levels of METH-2 in paired normal and tumour lung tissues. Patient numbers are shown. The heavier band represents the control gene (O28) and the lower band represents METH-2. A remarkable reduction of METH-2 transcript is observed in all tumour samples. N: normal; T: tumour.

**Figure 2:**
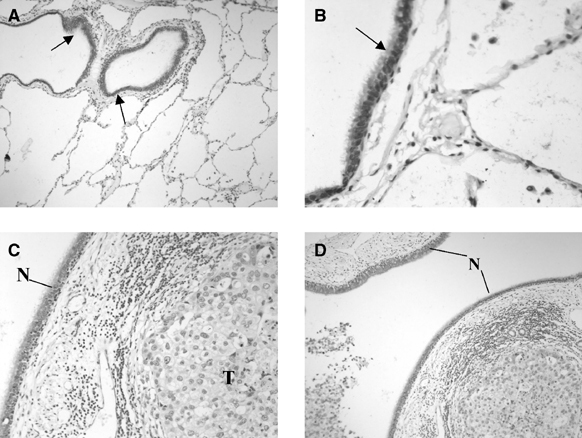
Immunohistochemical detection of METH-2 protein in lung tissues. (**A**, **B**) Normal lung. Positive staining is observed in ciliated bronchial epithelial cells (arrows). (**C**, **D**) Lung adenocarcinoma showing weakly positive tumour cells (T) and moderately positive staining of adjacent normal bronchial epithelium (N). Counterstained with H&E. Magnification: (**A**, **D**) × 10; (**B**, **C**) × 20.

Comparative multiplex RT–PCR (cmRT–PCR) analysis of METH-2 in 23 paired normal and lung cancer tissue cDNAs demonstrated a dramatic reduction of METH-2 transcript in 23 out of 23 (100%) lung tumour samples (Figure 1

). We have also undertaken IHC analysis in 20 NSCLC tissue sections (11 ADC and nine SCC) and 11 histologically normal adjacent lung sections. METH-2 protein is expressed in the bronchial epithelium and specifically in the cytoplasm of the ciliated cells (Table 1, Figure 2A and B

). Bronchial epithelium in adjacent normal tissue sections presented strong cytoplasmic staining in eight out of 11 (73%) samples, weak staining in two samples and negative staining in one sample. Interestingly, eight of the examined tumour sections contained some normal respiratory mucosa incidentally present within or adjacent to the tumour sample (listed in the ‘N_T_’ column of Table 1, also see Figure 2C and D). Six out of these eight showed strong and two weak staining. In contrast, only one out of 20 tumour section was strongly positive, nine were weakly positive and 10 were negative (Table 1, Figure 2C and D), which constitutes a significant difference compared to both the adjacent normal (Fisher's test, *P*=1.2 × 10^−4^) and the ‘internal’ normal (Fisher's test, *P*=3.8 × 10^−4^) areas.

In this small series, staining for METH-2 protein did not correlate with tumour histology, differentiation, TNM status or age of the patient.

### Methylation status of METH-2 promoter

**Figure 3:**
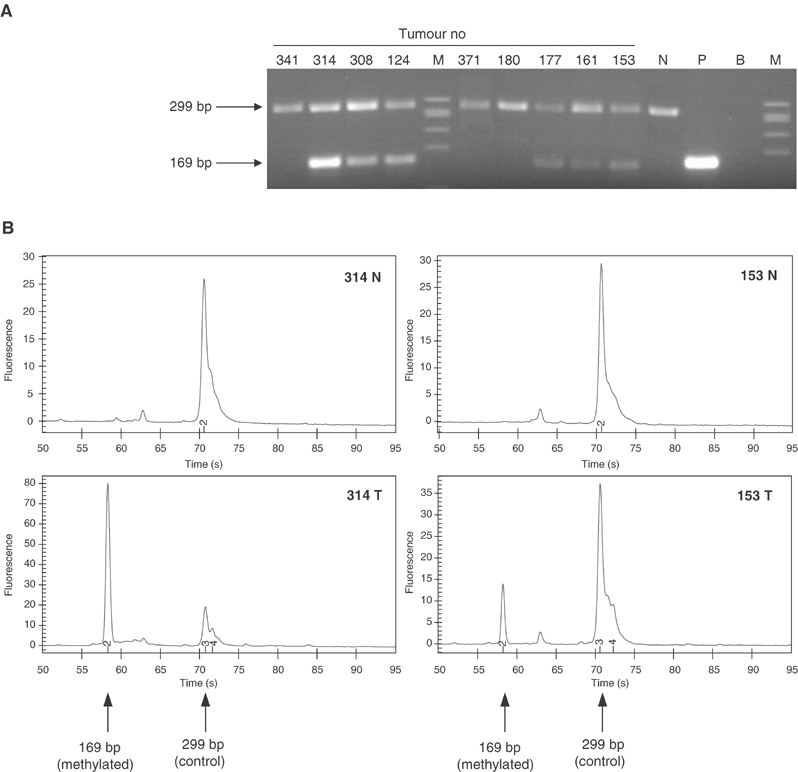
(**A**) METH-2 cMSP analysis on lung tumour DNAs. The presence of the methylation-independent control fragment (299 bp) confirms sufficient amount of converted DNA in the reaction, while the presence of the methylation-specific product (169 bp) demonstrates the existence of methylated copies. DNAs from tumours 341, 371 and 180 show no methylation of METH-2, while those from 314, 308, 124, 177, 161 and 153 demonstrate methylation of the METH-2 promoter. N: negative (unmethylated) control; P: positive (methylated with *Sss*I methylase) control; M: X174/*Hae*III. (**B**) Capillary electrophoretic analysis of METH-2 cMSP products on Agilent Bioanalyser 2100 DNA chip. The presence of the methylation-independent control peak (299 bp) indicates sufficient amount of converted DNA, while the presence of the methylation-specific product peak (169 bp) demonstrates the existence of methylated copies.

For the methylation analysis of the promoter region of METH-2, we included the samples used for expression analysis plus a further set of 27 available NSCLC DNAs, leading to a total of 50 NSCLC samples (24 ADC and 26 SCC). A total of 29 samples (58%) demonstrated hypermethylation of the METH-2 promoter (Figure 3

). METH-2 hypermethylation was more frequent in ADC (16 out of 24, 67%) than SCC (13 out of 26, 50%) although this difference is not statistically significant (Fisher's test, *P*=0.18). Methylation in normal tissue was found only in one case (sample L352) while the rest were negative. The methylation status of the METH-2 promoter did not correlate with age, differentiation or TNM status.

### Homozygous deletion and allelic imbalance analysis of METH-2

**Figure 4:**
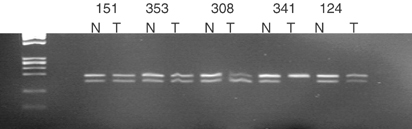
Allelic imbalance analysis of METH-2 using an intragenic SNP marker. Five heterozygotes are demonstrated. Sample pairs 151, 353 and 124 show no AI. Sample pairs 341 and 308 show AI in the tumour tissue (indicated by the disturbance of the allele ratios compared to the normal).

No homozygous deletions of METH-2 were identified in any lung cancer cell lines tested, with a clear PCR product amplifiable in each case. Allelic imbalance analysis of METH-2 using an RFLP assay for the rs1552330 intronic SNP demonstrated nine heterozygous and 14 homozygous (noninformative) samples. Allelic imbalance was observed in four out of nine (44%) of the informative samples (Figure 4

). Allelic imbalance was demonstrated as a change in the allele ratio between normal and tumour rather than a complete loss of one allele in tumour. Incomplete loss of an allele is likely to be due to the presence of normal contaminating cells in the tumour sections.

## DISCUSSION

Angiogenesis is regulated through a balance of angiogenic and antiangiogenic factors, and the angiogenic switch, which permits the expansion of a small neoplastic lesion, is a key event in tumour progression (Cox *et al*, 2000). Angiogenesis is also critically important in the context of the growth of disseminated micrometastases, which may conceivably be triggered by the surgical removal of a primary lesion (O'Reilly *et al*, 1999). In this study, we have validated the expression pattern of the antiangiogenic factor METH-2 in NSCLC following the identification of the tumour under-representation of its transcript in a microarray analysis of primary disease (Heighway *et al*, 2002). Furthermore, we have investigated plausible genetic and epigenetic phenomena such as allelic loss, homozygous deletions and promoter hypermethylation that may be responsible for this downregulation.

The sequence of METH-2 was originally identified during an attempt to find novel cDNAs with a thrombospondin type I (TSP-1) antiangiogenic domain (Vazquez *et al*, 1999). METH-2 is highly expressed in adult and fetal lung, and is found at lower levels in the brain, placenta, heart, stomach and fetal brain and kidney. Immunohistochemical detection of METH-2 protein in this study demonstrated strong cytoplasmic localisation in the differentiated bronchial epithelial cells of the majority of the normal samples.

In this study, METH-2 gene mRNA expression was dramatically reduced relative to histologically normal epithelial cells in all NSCLC tissues tested. Given that the tumours were randomly selected from our database, and were independent of those analysed in the initial microarray study, this downregulation is likely to represent a common state for neoplastic lung tissue. This global reduction of METH-2 transcript and protein in the lung tumour tissues could suggest either that the gene is abnormally downregulated in tumour providing a possible selective advantage, or alternatively that the low level of expression seen is more typical of the tumour progenitor cell.

Concerning the possible causes of METH-2 silencing, interestingly, more than 50% of tumours demonstrated the presence of hypermethylated alleles in the gene's promoter region, with the majority (49 out of 50) of normal samples showing no methylation. These results suggest that DNA methylation may be an important mechanism of transcriptional inactivation of METH-2 in NSCLC. The MSP approach we used is not quantitative; therefore, no conclusions can be currently drawn regarding the percentage of cells carrying methylated alleles. Further investigations using a real-time or cloning and sequencing approach are required to clarify this issue and demonstrate the precise impact of methylation on METH-2 silencing. We have also shown in a small number of cases using an internal SNP that allelic loss occurred in four out of nine informative samples. The use of additional SNPs and/or microsatellites in the region providing higher percentage of heterozygotes is required in future studies to confirm this initial observation.

Comparative multiplex RT–PCR analysis of METH-2 demonstrated no discernable expression in the 23 specimens under investigation, thus no valid associations could be made between the presence of DNA methylation and mRNA expression. Similarly, with only one out of 20 tumours showing strong expression of the METH-2 protein, no association between DNA methylation and METH-2 protein expression could be made. Nonetheless, it appears that METH-2 silencing is universal in NSCLC due to a number of reasons, which include DNA methylation and AI-associated abnormalities. However, a larger series of samples must be investigated to verify this hypothesis. We have not observed homozygous deletions of METH-2 in a panel of lung cancer cell lines, suggesting that this phenomenon may be infrequent in NSCLC.

The protein levels in tumours were low in 19 out of 20 samples and this observation is consistent with the observed mRNA transcript downregulation. It is noteworthy that although a METH-2 transcript was diminished in all tumours by RT–PCR, nine out of 20 tumours demonstrated reduced but not negative expression. Also, in certain instances, normal bronchial epithelium within the tumour sections stained positive in these samples. It is likely that the transcript copy number in such samples was too low to be detected under the RT–PCR conditions used. We must also consider the possibilities that (a) depending on the half-life in the tumour cell, protein may be present in the absence of amplifiable transcript, and (b) that any unmatched IHC and RT–PCR results could be due to the topographically different section of the tissue samples used for each procedure.

Immunohistochemical analysis revealed three normal adjacent epithelium areas (in patients L323, L336 and L341; see Table 1) with apparently reduced protein expression but clearly detectable levels of mRNA. One possibility is that post-transcriptional modification may be responsible for the apparent reduction of observed protein levels in these cells. This is also supported by the fact that methylation, which appears to be a significant cause of silencing in tumours, was only observed in a single normal sample. Three tumour samples (L352, L308 and L314) showed protein expression and also promoter hypermethylation (see Table 1). It is likely that METH-2 hypermethylation in these cases was partial and not sufficient to silence the gene completely.

The downregulation of expression seen in normal epithelium poses a question on the timing and significance of such events. There were no data in this study to indicate more frequent or greater loss of METH-2 expression in advanced disease. However, all the samples examined for expression and the vast majority of samples examined for methylation were staged as T2, minimising the chance of statistically valid conclusions. In addition, no metaplastic or dysplastic sites were observed in the sections examined; thus, the status of METH-2 in lung preneoplasia still remains to be clarified.

In conclusion, we have highlighted METH-2 as a gene that is dramatically downregulated in NSCLC. In many cases, promoter hypermethylation and AI appear to have an effect on this. The striking downregulation observed in all tumours examined and the potential of the gene product as an antiangiogenic therapeutic agent certainly make it worthy of further investigation.
